# Controlling extrudate volume fraction through poroelastic extrusion of entangled looped fibers

**DOI:** 10.1038/s41467-023-36860-y

**Published:** 2023-03-04

**Authors:** Zehao Pan, Janine K. Nunes, Camille Duprat, Ho Cheung Shum, Howard A. Stone

**Affiliations:** 1 Department of Mechanical and Aerospace Engineering, Princeton University, Princeton, NJ 08544 USA; 2 Department of Mechanical Engineering, University of Hong Kong, Pokfulam Road, Hong Kong, 999077 China; 3 Present Address: LadHyX, CNRS, Ecole polytechnique, Institut polytechnique de Paris, 91120 Palaiseau, France

**Keywords:** Biomedical materials, Mechanical engineering, Fluid dynamics

## Abstract

When a suspension of spherical or near-spherical particles passes through a constriction the particle volume fraction either remains the same or decreases. In contrast to these particulate suspensions, here we observe that an entangled fiber suspension increases its volume fraction up to 14-fold after passing through a constriction. We attribute this response to the entanglements among the fibers that allows the network to move faster than the liquid. By changing the fiber geometry, we find that the entanglements originate from interlocking shapes or high fiber flexibility. A quantitative poroelastic model is used to explain the increase in velocity and extrudate volume fraction. These results provide a new strategy to use fiber volume fraction, flexibility, and shape to tune soft material properties, e.g., suspension concentration and porosity, during delivery, as occurs in healthcare, three-dimensional printing, and material repair.

## Abstract (web summary)

When a suspension of particles passes through a constriction the particle volume fraction either decreases or remains the same. Pan et al. report that an entangled fiber suspension increases its volume fraction greater than a factor of 10 after passing through a constriction.

## Introduction

Injectable biomaterials are used widely as cavity-filling agents, such as in the treatments of aneurysms^1^, spinal cord regeneration^2^, and wound dressings^3,4^. In these applications, it is desirable during the delivery that the material should have good flowability to facilitate movement in a thin catheter or needle. After delivery, the material should transition rapidly to poor flowability to prevent egress. Suspensions have emerged as an attractive candidate for this class of materials^5^, whose rheological response is coupled to the local volume fraction. However, it remains a challenge to create a suspension with low concentration during material delivery to facilitate flow and high concentration at the delivered site to ensure material localization and functionality.

When pushed through a constriction, a dense particulate suspension made of spherical or near-spherical particles can suffer from “demixing"^6^, “liquid migration”^7,8^, “self-filtration”^9,10^, or “dilatancy”^11^, where the liquid moves relative to the solid phase, producing a more dilute mixture at the exit of a constriction. So far, most research on the extrusion of suspensions has been focused on particulate systems.

However, suspensions made from fibers can be mechanically distinct from their spherical and near-spherical counterparts in their ability to respond elastically to tensile stresses. In the absence of permanent linkages, entanglements among different fibers can arise from static friction, irregular shapes, and interlocking structures^12–15^. When the density of these linkages reaches a threshold, the suspension starts to respond as a soft elastic material^16^. These entanglements offer the fiber network stability under moderate shear or extensional flow conditions^17^, where the elasticity of such entangled fiber networks has been studied using simulations^16,18,19^. Microscopically, entanglements in an athermal fiber network have been related to the number of ‘stable’ contacts per fiber during loading, yet a coherent picture of the rheological properties of the various entangled systems above is lacking due to poor mechanistic understanding^20^. Currently, there is a lack of experimental studies and insights on the permeability and elasticity of entangled fiber suspensions due to difficulty in measurements. As a result, their volume fraction variations are not fully understood and the dynamical responses in a flow field we report here have not been recognized previously.

When used as a biomaterial, fiber suspensions offer several unique properties. The fibers can be readily made from existing biocompatible materials for toxicity-sensitive applications using established fiber spinning methods^21^. A loose packing of fibers can also create a gel with hierarchical porosity where the pore sizes range from the scale of molecular cross links in the fiber to the typical distance between fibers in a suspension^22,23^. Hence, from a material design perspective, in order to control the mechanical and transport properties, it is important to understand how the volume fraction of such a soft elastic material changes upon extrusion.

In this paper, we experimentally characterized the flow field and volume fraction variations when suspensions of designed flexible, micro-textured, and entangled microfibers pass through a constriction. As the microfibers enter the constriction, instead of the commonly observed “self-filtration”, the faster moving fibers in the constriction cause elastic stretching among the entangled fibers upstream of the constriction. As a result, the suspension downstream of the constriction has an increased volume fraction. We model the process based on a poroelastic framework that takes into account the coupling between the elastic deformation and the flow within the network of entangled fibers. Our work may also inspire modeling of similar processes such as the injection of granular materials with dynamic bonds^24^ and biologically active networks^25^.

## Results

### Fiber suspensions passing a constriction

The poly(ethylene glycol) diacrylate (PEGDA) fibers were made using a jet-assisted wet spinning (JAWS) method described in Methods. A custom-made transparent channel with rectangular cross section (8 mm in depth by 10 mm in width) was used to visualize the microfiber suspensions as they pass through a constriction.

**Fig. 1:**
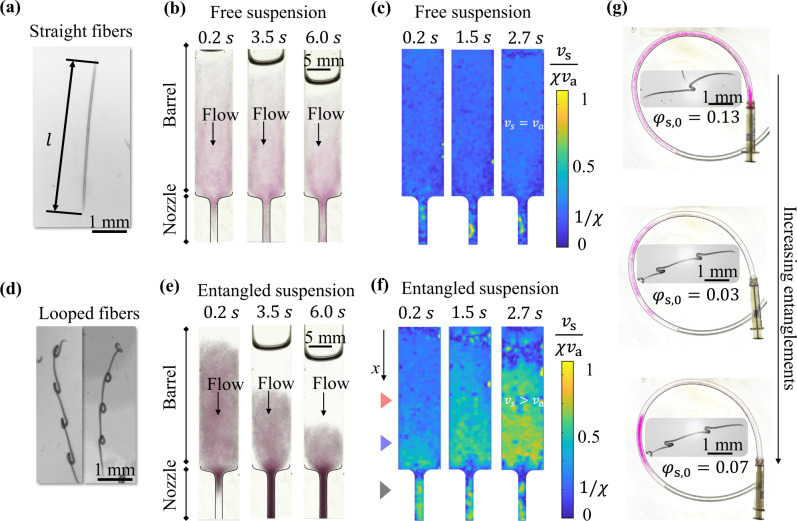
Concentration and velocity variations when suspensions of straight and looped fibers pass a constriction. **a**, **d** Shapes of the straight and looped fibers, respectively. **b**, **e** Snapshots of suspensions of **a** straight and **d** 4-looped fibers flowing through a constriction when a constant flow rate of 8 ml min^−1^ is applied. The initial fiber volume fractions *ϕ*_s,0_ = 0.2 for both cases. **c**, **f** Velocity (*x* component) distributions of the straight and looped fibers from PIV measurements of the experiments in, respectively, **b**, **e** at three different times. The fiber velocity *v*_s_ is normalized by the average velocity in the nozzle *χv*_a_. The colored triangles to the left of **f** are positions measured in Fig. 3b. **g** Distribution of magenta fiber extrudate in a tubing after extrusion from a 3 ml syringe at a flow rate of 5 ml min^−1^. The 1- and 2-looped fibers are shown in the center along with initial volume fractions *ϕ*_s,0_ before the extrusion.

We first tested the extrusion of stiff straight fibers with diameter *d* = 60 μm, length *l* = 4.3 mm, and aspect ratio *l*/*d* = 72 (Fig. 1a). Before the experiment, a suspension of straight fibers was introduced in an open barrel with an initial volume fraction of *ϕ*_s,0_ = 0.2. At *t* = 0 s, a constant withdrawal flow rate *Q*_0_ = 8 ml min^−1^ was applied through a syringe at the end of the nozzle, which drew the suspension from the barrel into the nozzle. Snapshots of the experiment are shown in Fig. 1b. The fibers in the barrel moved with the surrounding fluid without being dragged into the nozzle by the other fibers and the volume fraction in the nozzle was largely unchanged. Thus, the suspensions made from stiff straight fibers are referred to as free suspensions. The magnitude of the *x* velocity component of the fibers *v*_s_ is quantified in Fig. 1c using particle image velocimetry (PIV)^26^. The specified flow rate is translated into an average velocity *v*_a_ ≡ *Q*_0_/*A*_b_ in the barrel, where *A*_b_ is the cross sectional area of the barrel. The average velocity in the nozzle is *χv*_a_, which is controlled by a geometric parameter *χ* ≡ *A*_b_/*A*_n_, where *A*_n_ is the cross sectional area of the nozzle. For the results in Fig. 1c, *χ* ≡ *A*_b_/*A*_n_ = 5. The fibers in the nozzle moved at a velocity χ^*^ times faster than *v*_a_. PIV results indicate χ^*^ ≈ *χ* throughout the extrusion process. In the barrel, the velocity *v*_s_ of the straight fibers remained close to *v*_a_.

Entanglements within fiber network have been associated strongly with the friction coefficient between the fibers^20^. To enhance the entanglements among the low-friction hydrogel fibers, we added mechanical oscillation to the JAWS fabrication method to make looped fibers with an average diameter *d* = 60 μm, length *l* = 4.5 mm and aspect ratio *l*/*d* = 75 (Fig. 1d). Each looped fiber has four permanent non-intersecting loops that have a typical dimension of 200 μm. Based on our experimental observations and imaging, the loops act like mechanical linkages that prevent sliding and effectively create transient joints among the fibers (Supplementary Fig. S2). The multiple loops on a single fiber allow a network of mechanical linkages to form. Thus, the suspensions made from looped fibers are referred to as entangled suspensions. Similar to the free suspension, the same extrusion experiment is carried out under the same flow rate and initial fiber volume fraction (Fig. 1e). For the suspension of looped fibers, the faster moving fibers in the nozzle were entangled with the fibers in the barrel. As a result, the fibers became more concentrated in the nozzle, as evident from the darker color in the images, and left excess water behind in the barrel. The magnitude of the *x*-velocity component of the fibers *v*_s_ is quantified in Fig. 1f. The PIV results also indicate χ^*^ ≈ *χ* for looped fibers. The observed response is different from the stiff straight fibers, as the looped fibers in the barrel gradually accelerated from *v*_a_ to *χv*_a_.

The approximation χ^*^ ≈ *χ* requires sufficiently high flow rates *Q*_0_ and is one of the operating conditions when extruding a fiber suspension through a constriction. “Self-filtration” occurred when *Q*_0_ < 5 ml min^−1^. For a flow rate *Q*_0_ ≈ 5 ml min^−1^, the fibers started flowing intermittently with χ^*^ < *χ*. In particular, in the above setup when *Q*_0_ was reduced to 1 ml min^−1^, χ^*^ = 0, the fibers clogged before the constriction and were unable to enter the nozzle until most of the liquid had left the barrel. The clogging regime occurs due to a combination of fiber interactions with the wall, fiber stiffness, and the three-dimensional deformations at the constriction^27,28^, which are beyond the scope of this study. While such clogging or partial clogging are regularly observed for particles flowing through a constriction, the high flow rate regime where the extruded suspension is concentrated is unique to the entangled fibers. For the rest of the paper, we will thus focus on this regime where the fibers move at the local average velocity in the nozzle, i.e., χ^*^ = *χ*.

Different fiber geometries affect the fiber entanglements that produce different extrusion regimes. We first made 1- and 2-looped fibers using the same fabrication setup. Different extrudate morphologies for the number of loops and volume fractions are displayed in Fig. 1g after all the initial volume in a syringe was extruded into tubing. Two morphologies can be observed: the extrudates are either dispersed and fragmented, or form an integral ‘gel’. The formation of this integral mass is related to the existence of long-range fiber-fiber interactions that depend on the capacity of the fibers to entangle, which is controlled by their shape and flexibility. We found at least two loops are needed for looped fibers to form a ‘gel’. For straight fibers, various *l*/*d* up to 360 were tested. Based on our experiments with a fixed barrel length, an integral ‘gel’ can only form at high aspect ratios, when *l*/*d* = 360, where *l* is more than 50% of the barrel length (Supplementary Fig. S3). For the rest of the paper we focus on the 4-looped fibers as a model material for their shorter length compared to the barrel length and strong entanglements at low volume fractions.

### Poroelastic model for the extrusion of an entangled suspension

As experimentally documented in the previous section, entangled suspensions respond to velocity variations at a constriction differently from free suspensions. To understand the mechanical response of an entangled fiber suspension under extensional deformations, we pulled a few fibers from a suspension of fibers while monitoring the pull-out force. The calculated stress-strain relationship is shown in Supplementary Fig. S4. As fibers were pulled from the entangled suspension, a much higher stress was registered compared to similar measurements made for suspensions of straight fibers. For short stiff fibers, i.e., the free suspension, no stress can be measured, indicating the absence of entanglements. The results also showed that the looped fiber suspension overall had a linear response to stretching before the entanglements failed.

**Fig. 2:**
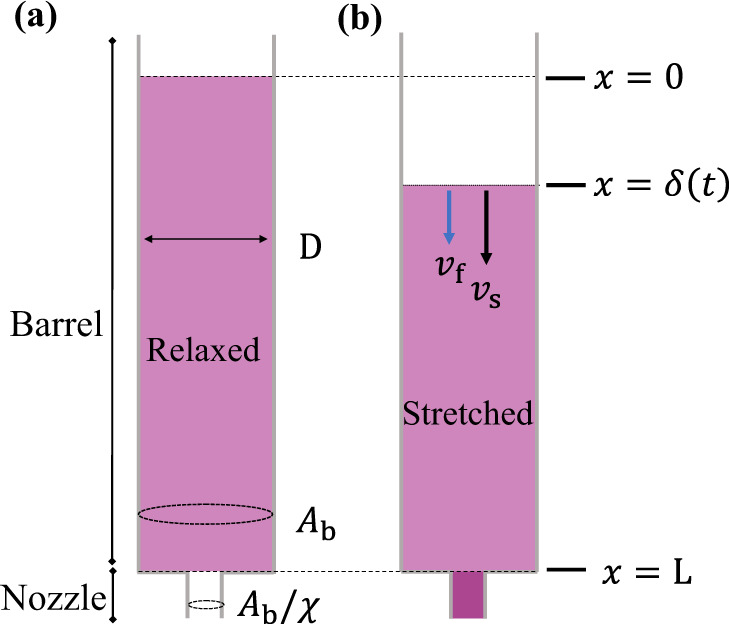
Poroelastic model for the extrusion of entangled fibers in a long tube. **a** The solid phase is laterally confined and relaxed in the barrel (length *L*). The barrel cross section has typical width *D*. The ratio of the cross-sectional areas between the barrel and nozzle is *χ*, where *χ* > 1. **b** When a constant total flux *v*_a_ is applied from the barrel to the nozzle, the fluid phase adopts velocity *v*_f_(*x*, *t*) and the solid phase *v*_s_(*x*, *t*). The displacement of the free end of the suspension is denoted by *x* = *δ*(*t*), with *δ*(0) = 0. At *x* = *L*, the solid velocity *v*_s_ increases to *χv*_a_, stretching the solids in the barrel.

Based on the above observations and measurements, we propose a uniaxial poroelastic extrusion model for a long barrel, as sketched in Fig. 2. Initially, a porous material is in a relaxed state in the barrel of an extrusion setup spanning distance *L* from the constriction at *x* = *L* (Fig. 2a). The unconfined boundary of the solid phase at *x* = 0 is free of stress in the *x* direction. The material has uniform porosity, defined as the volume fraction of the fluid *ϕ*_f,0_ = 1 − *ϕ*_s,0_. From the PIV measurements in Fig. 1, the entangled suspension maintains an approximately plug flow during extrusion. Thus the channel wall is assumed to be frictionless. Starting from *t* = 0, a total flow rate *Q*_0_ is then applied, creating an average velocity *v*_a_ in the barrel that is a function of the fluid velocity *v*_f_ and the solid velocity *v*_s_ (Fig. 2b). The entrance flow near the constriction is assumed to occupy a region *D* ≪ *L* (long barrel approximation). As the suspension enters the nozzle (*x* = *L*), it moves at speed *χv*_a_, resulting in the stretching of the entangled solids in the barrel. The flow is approximated as one dimensional in the domain between 0 ≤ *x* ≤ *L*. The equations for uniaxial poroelastic deformation have been stated elsewhere^29,30^ and will be described briefly here.

In the Eulerian (laboratory) frame, the displacement field of the solid at time *t* is *u*_s_ = *x* − *X*(*x*, *t*), where *X* is the reference position of the material. The solid deformation changes the solid volume fraction. In the uniaxial geometry with uniform initial porosity, the deformation gradient ${(1-\frac{\partial {u}_{{{{{{{{\rm{s}}}}}}}}}}{\partial x})}^{-1}$ is equal to the volume fraction variation $\frac{1-{\phi }_{{{{{{{{\rm{f}}}}}}}},0}}{1-{\phi }_{{{{{{{{\rm{f}}}}}}}}}}$, which leads to a relationship between *u*_s_ and the time-varying porosity field *ϕ*_f_(*x*, *t*), i.e.,

1 $$\frac{\partial {u}_{{{{{{{{\rm{s}}}}}}}}}}{\partial x}=\frac{{\phi }_{{{{{{{{\rm{f}}}}}}}}}-{\phi }_{{{{{{{{\rm{f}}}}}}}},0}}{1-{\phi }_{{{{{{{{\rm{f}}}}}}}},0}}.$$

A more rigorous derivation in tensor form can be found in ref. ^29^. The corresponding velocity of the solid is the material derivative of the displacement field: ${v}_{{{{{{{{\rm{s}}}}}}}}}=\frac{D{u}_{{{{{{{{\rm{s}}}}}}}}}}{Dt}=\frac{\partial {u}_{{{{{{{{\rm{s}}}}}}}}}}{\partial t}+{v}_{{{{{{{{\rm{s}}}}}}}}}\frac{\partial {u}_{{{{{{{{\rm{s}}}}}}}}}}{\partial x}$, yielding ${v}_{{{{{{{{\rm{s}}}}}}}}}=\frac{\partial {u}_{{{{{{{{\rm{s}}}}}}}}}}{\partial t}/(1-\frac{\partial {u}_{{{{{{{{\rm{s}}}}}}}}}}{\partial x})$.

The total flux in the barrel *v*_a_ represents the total volume flow per area per time, which is an imposed constant value in our experiments, and is divided among the fluid velocity *v*_f_(*x*, *t*) and the solid based on their volume fractions (*ϕ*_f_ is the fluid volume fraction):

2 $${v}_{{{{{{{{\rm{a}}}}}}}}}\equiv {\phi }_{{{{{{{{\rm{f}}}}}}}}}{v}_{{{{{{{{\rm{f}}}}}}}}}+(1-{\phi }_{{{{{{{{\rm{f}}}}}}}}}){v}_{{{{{{{{\rm{s}}}}}}}}}.$$

We assume the fluid flows relative to the solid according to Darcy’s law^31^:

3 $${\phi }_{{{{{{{{\rm{f}}}}}}}}}({v}_{{{{{{{{\rm{f}}}}}}}}}-{v}_{{{{{{{{\rm{s}}}}}}}}})=-\frac{k({\phi }_{{{{{{{{\rm{f}}}}}}}}})}{\mu }\frac{\partial p}{\partial x},$$

where *k*(*ϕ*_f_) is the porosity-dependent permeability, *μ* the viscosity of fluid, and *p* the pressure within the suspension. The continuity equation for the fluid is

4 $$\frac{\partial {\phi }_{{{{{{{{\rm{f}}}}}}}}}}{\partial t}+\frac{\partial }{\partial x}({\phi }_{{{{{{{{\rm{f}}}}}}}}}{v}_{{{{{{{{\rm{f}}}}}}}}})=0.$$

Combining Eqs. (2)–(4) we have a one-dimensional poroelastic equation:

5 $$\frac{\partial {\phi }_{{{{{{{{\rm{f}}}}}}}}}}{\partial t}+\frac{\partial }{\partial x}\left({\phi }_{{{{{{{{\rm{f}}}}}}}}}{v}_{{{{{{{{\rm{a}}}}}}}}}-(1-{\phi }_{{{{{{{{\rm{f}}}}}}}}})\frac{k({\phi }_{{{{{{{{\rm{f}}}}}}}}})}{\mu }\frac{\partial p}{\partial x}\right)=0.$$

The total stress *σ* within the suspension is composed of the elastic stress of the entangled fiber network ${\sigma }_{xx}^{{\prime} }$ and hydrodynamic pressure *p*: ${\sigma }_{xx}={\sigma }_{xx}^{{\prime} }-p$. Neglecting inertia and in the absence of body forces, the effective stress ${\sigma }_{xx}^{{\prime} }$ (or “Terzaghi stress”^32^) in the elastic fiber network satisfies

6 $$\frac{\partial {\sigma }_{xx}^{{\prime} }}{\partial x}=\frac{\partial p}{\partial x}.$$

Because the Young’s modulus of the crosslinked PEGDA used in the experiments is much larger than the effective Young’s modulus of the fiber network (100 kPa versus 100–1000 Pa), the deformations are assumed to originate solely from bending and rearrangements of the fibers. Based on the results from our pull-out tests, we use a linear elastic relation (Hooke’s law) between the deformation and the entangled fiber stress, ${\sigma }_{xx}^{{\prime} }$:

7 $${\sigma }_{xx}^{{\prime} }={E}_{{{{{{{{\rm{eff}}}}}}}}}\frac{\partial {u}_{{{{{{{{\rm{s}}}}}}}}}}{\partial x},$$

where *E*_eff_ is the effective Young’s modulus of the suspension.

The permeability *k*(*ϕ*_f_) is assumed to vary from its initial relaxed state *k*_0_ similar to a randomly oriented suspension of fibers^17^:

8 $$\frac{k({\phi }_{{{{{{{{\rm{f}}}}}}}}})}{{k}_{0}}=\frac{1-{\phi }_{{{{{{{{\rm{f}}}}}}}},0}}{\ln (1-{\phi }_{{{{{{{{\rm{f}}}}}}}},0})+0.931}\frac{\ln (1-{\phi }_{{{{{{{{\rm{f}}}}}}}}})+0.931}{1-{\phi }_{{{{{{{{\rm{f}}}}}}}}}},$$

which is valid for *ϕ*_f,0_ > 0.7.

At *x* = *L*, we assume *v*_s_ = *χv*_a_, which neglects the entrance flow region based on the long-barrel approximation. The above equations are to be solved for *u*_s_, where *v*_s_ = *χv*_a_ serves as a Neumann boundary condition for the solid phase at *x* = *L*. The velocity at *x* = *δ*(*t*) is to be calculated.

Nondimensionalization of Eq. (5) yields the poroelastic time scale for the dynamical response,

9 $${T}_{{{{{{{{\rm{pe}}}}}}}}}=\frac{\mu {L}^{2}}{{E}_{{{{{{{{\rm{eff}}}}}}}}}{k}_{0}}.$$

The model is controlled by three independent dimensionless parameters, i.e., the strength of the imposed total flux that compares the flow time scale *T*_f_ = *L*/*v*_a_ to the poroelastic time scale

10 $${\overline{v}}_{a}=\frac{{v}_{{{{{{{{\rm{a}}}}}}}}}\mu L}{{E}_{{{{{{{{\rm{eff}}}}}}}}}{k}_{0}}=\frac{{T}_{{{{{{{{\rm{pe}}}}}}}}}}{{T}_{{{{{{{{\rm{f}}}}}}}}}},$$

the cross sectional area ratio *χ*, and the initial porosity *ϕ*_f,0_. The equations are integrated using a Runge-Kutta scheme (adopted from ref. ^29^) in a moving boundary domain over the entire time the suspension spends in the barrel.

### Comparison between model and experiments

**Fig. 3:**
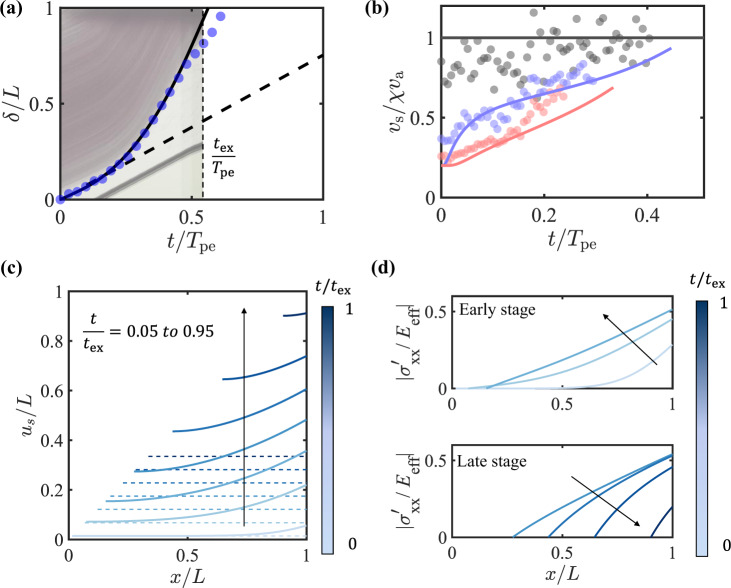
Poroelastic model simulations for the extrusion of looped fiber suspensions, as shown in Fig. 1e, f. Actual experimental parameters and a single fitting parameter *F* ≡ *k*_0_*E*_eff_ = 91 nN are used for the simulation, resulting in ${\overline{v}}_{a}=0.63$. **a** The computed (solid line) and experimentally measured (dots) free boundary displacement *δ*(*t*) of the suspension during extrusion. The dot size represents typical errors in the measurement. The inclined dashed line is the trajectory at constant velocity *v*_a_. Kymograph of the center line of the setup is presented in the background. At time *t*_ex_, all solid material has passed the constriction. The poroelastic time *T*_pe_ is defined in Equation (9). **b** Comparison between the computed velocity profiles and measured PIV results at locations in Fig. 1f indicated by the corresponding colored triangles. Gray: nozzle (*x*/*L* = 1); blue: near constriction (*x*/*L* = 0.75); red: farther from constriction (*x*/*L* = 0.4). **c** The distribution of the normalized displacement field *u*_s_/*L* at times up to approximately *t*_ex_ (solid lines) and the displacement field without elasticity (dashed lines) over the same time domain. Time interval between each line is 0.15*t*_ex_. Darker color represents later times. **d** The normalized elastic stress ${\sigma }_{xx}^{{\prime} }/{E}_{{{{{{{{\rm{eff}}}}}}}}}$ at early (top panel) and late stages (bottom panel) of the extrusion.

To model the displacement and velocity variations during the extrusion of looped fibers, we compute the poroelastic model using the experimental values from Fig. 1 and a fitting parameter *F* ≡ *k*_0_*E*_eff_. Physically, *F* can be understood as the average force on a typical pore area to induce 100 % strain of the elastic network. Alternatively, *F*/*μ* measures the ‘diffusivity’ or ‘coefficient of consolidation’ of the network^33^. In Fig. 3a we compare the computed displacement of *δ*(*t*) with the experimental results of the boundary of the fiber-rich region. The experimentally measured *δ*(*t*) initially moved at constant speed *v*_a_, then accelerated for the rest of the extrusion. Using *F* = 91 nN, the simulated *δ* shows good agreement with the time variation of the experimental measurements. In comparison, paper pulp has a typical *F* = 30 nN at the same solid volume fraction^34^. All of the solid material has left the barrel by the time *t*_ex_, which yields an average velocity *L*/*t*_ex_ = 2.6*v*_a_. The deviation of the data from the fitted line, especially approaching *t*_ex_, is due to the fibers coming in contact with the syringe at the end of the nozzle.

Using the same *F*, we next compare the velocity fields between the experiments and simulations. In Fig. 3b, the measured velocities at the positions indicated by the colored triangles from Fig. 1f are compared with the simulation results at the same positions. The experimental velocity of the solid in the nozzle is close to *χv*_a_ despite variations in the PIV measurements due to limited lighting in the nozzle. Near the constriction (blue), the solid velocity starts from *v*_a_ at *t* = 0, then increases rapidly, followed by a more gentle increase, which is a feature observable in both the experiments and the simulations. The velocity increases at all positions in the barrel, but the rate of increase is higher near the constriction (blue versus red). Overall, the simulation captures the velocity evolution observed in the experiments both qualitatively and quantitatively.

Based on these validations of the model, Fig. 3c shows the displacement fields that are not readily observable in the experiments, which we compare with the displacement fields of a free suspension over the course of *t*_ex_. In a free suspension, *E*_eff_ = 0 Pa and the corresponding displacement field is uniform (flat) for all *x*. Without elastic stretching, the solids in the suspension across the domain will move at *v*_a_. The difference between the two cases are obvious from the beginning of the extrusion. For the entangled suspension, the displacement field exceeds the free suspension at *x* = *L*, where the velocity difference between the solid and the fluid is the greatest. The difference in displacement then expands over time throughout the suspension.

In contrast to the flat displacement field in a free suspension, entanglements create a displacement gradient that translates into the strain and stress within the fiber network. Figure 3d shows the internal elastic stress field during extrusion up to a time close to *t*_ex_. At *x* = *L*, the stress starts to increase at *t* = 0, then peaks in the middle of the extrusion process. At other locations, the onset of stress increase occurs at a later time. The highest stretching of the elastic network occurs at *x* = *L* and is less than 60% throughout the extrusion process. As a result of the linear relationship between the fluid volume fraction and the elastic stress from Eqs. (1) and (7), *ϕ*_f_ has similar dynamics to ${\sigma }_{xx}^{{\prime} }$ (Supplementary Fig. S5).

### Extrudate volume fraction of entangled fiber suspensions

**Fig. 4:**
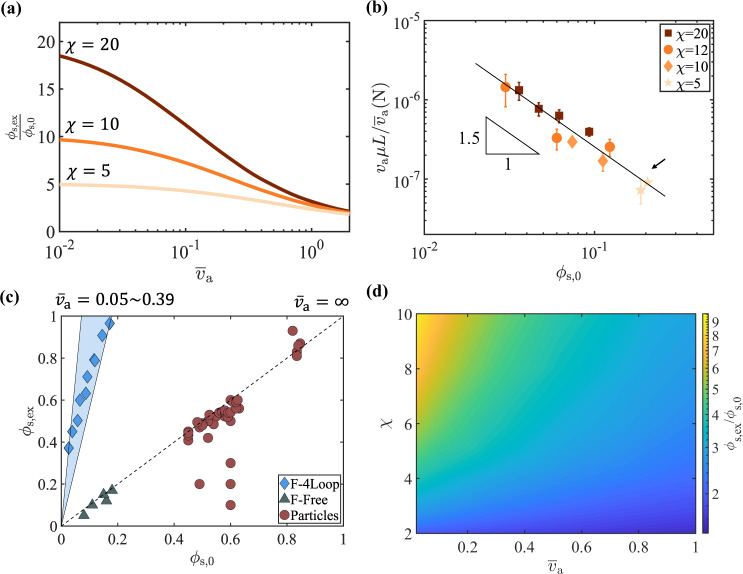
Extrudate volume fraction *ϕ*_s,ex_ as a function of ${\overline{v}}_{a}$ and *χ*. **a** Computed ratio between the extrudate and initial volume fractions as a function of ${\overline{v}}_{a}$ at three different *χ* values. These relationships are used to calculate ${\overline{v}}_{a}$ using experimentally measured *ϕ*_s,ex_/*ϕ*_s,0_. Displayed curves use *ϕ*_s,0_ = 0.05. **b** Experimentally fitted ${F}_{\exp }$ as a function of *ϕ*_s,0_ at different *χ* values. ${F}_{\exp }$ is calculated from ${\overline{v}}_{a}$ using ${F}_{\exp }={v}_{{{{{{{{\rm{a}}}}}}}}}\mu L/{\overline{v}}_{a}$. The error bars are calculated from five independent measurements. The experimental data of looped fibers from Fig. 1e is indicated by the black arrow. All experiments satisfy the condition *ϕ*_f_ > 0.7 required in Eq. (8). **c** Extrudate volume fractions as a function of initial volume fractions for different suspension materials. Circles are reported extrusion results from particle pastes made of ceramics^35 -- 40^ and polymers^7 --10,41^. The diamonds and triangles represent individual experiments performed with looped and straight fibers shown in Figs. 1b and e, respectively. The blue shaded region represents model predictions for a range of ${\overline{v}}_{a}$; the dotted line represents *ϕ*_s,ex_ = *ϕ*_s,0_ when *χ* = 1 or ${\overline{v}}_{a}=\infty$. **d** Computed *ϕ*_s,ex_/*ϕ*_s,0_ based on the poroelastic extrusion model as a function of *χ* and ${\overline{v}}_{a}$ at *ϕ*_s,0_ = 0.1.

After being extruded through a constriction, the entangled fiber suspension changes its volume fraction significantly. We define *ϕ*_s,ex_ as the average extrudate volume fraction calculated based on the total extrusion time ${\phi }_{{{{{{{{\rm{s,ex}}}}}}}}}\equiv \frac{L}{{v}_{{{{{{{{\rm{a}}}}}}}}}{t}_{{{{{{{{\rm{ex}}}}}}}}}}{\phi }_{{{{{{{{\rm{s}}}}}}}},0}$. Using the poroelastic extrusion model above, we can compute *ϕ*_s,ex_ as a function of ${\overline{v}}_{a}$ and *χ*. The results in Fig. 4a show how *ϕ*_s,ex_/*ϕ*_s,0_ varies with ${\overline{v}}_{a}$ for different values of *χ*. The third independent variable in the model *ϕ*_s,0_ has little effect on *ϕ*_s,ex_ (Supplementary Fig. S5). Given the definitions, 1≤*ϕ*_s,ex_/*ϕ*_s,0_ ≤ *χ*, where the variations depend on ${\overline{v}}_{a}$: thus, the stronger the entangled network (smaller ${\overline{v}}_{a}$), the higher *ϕ*_s,ex_.

We experimentally extruded looped fiber suspensions and measured *ϕ*_s,ex_ at different conditions of *A*_b_, *χ*, *ϕ*_s,0_, *Q*_0_ and *L* (Supplementary Fig. S6 and Table 1). These measurements are fitted to the computational results by adjusting ${\overline{v}}_{a}$. Using measurable dimensional quantities *v*_a_, *L* and *μ*, the fitted ${\overline{v}}_{a}$ thus allows the calculation of ${F}_{\exp }\equiv {v}_{{{{{{{{\rm{a}}}}}}}}}\mu L/{\overline{v}}_{a}$ based on experiments. While *k*_0_ and *E*_eff_ are functions of *ϕ*_s,0_, their specific relations are determined by the microstructure and entanglements of the suspension that are unknown at this point^42^. Given the same fiber geometry, however, their product *F* would have a universal relationship with *ϕ*_s,0_, independent of the extrusion conditions.

The calculated ${F}_{\exp }$ is shown in Fig. 4b as a function of *ϕ*_s,0_. The data show the relationship between ${F}_{\exp }$ and *ϕ*_s,0_ are conserved when different *χ*, *L*, *v*_a_ and *A*_b_ are used. The fitted power law has an exponent of −1.5 and R^2^ value of 0.95. Rheological measurements of the shear modulus ${G}^{{\prime} }$ and yield stress of the looped fiber suspension show a power law relationship with *ϕ*_s,0_ with exponents of 3.0 and 3.6, respectively (Supplementary Fig. S7). If *E*_eff_ follows the same power law as ${G}^{{\prime} }$ or the yield stress, *k*_0_ would scale as *ϕ*_s,0_ to the power of −4.5 or −5.1, similar to that of pulp suspensions and distinct from a suspension of random rods^43^ in the same range of *ϕ*_s,0_. Another method to independently verify ${F}_{\exp }$ as an estimation of *F* is through the direct measurement of *E*_eff_. From the pull-out test, *E*_eff_ is estimated to be on the order of 10^1^ to 10^2^ Pa at *ϕ*_s,0_ = 0.15 (Supplementary Fig. S4). We can estimate *k*_0_ using Jackson and James’ formula for randomly packed fibers^17^, which results in a value of *k*_0_ ~ 3 × 10^−9^ m^2^. This provides an estimate for *k*_0_*E*_eff_ between 3 × 10^−8^ N and 3 × 10^−7^ N, in agreement with the value obtained using the extrusion experiments presented in Fig. 4b. These results demonstrate ${F}_{\exp }$ is a reasonable characterization of *F* using extrusion experiments.

Many other factors could contribute to the variations of ${F}_{\exp }$ reported in Fig. 4b. In our continuum treatment of the fiber suspension, *F* takes into account the unique microstructure including the pore size for *k*_0_ and entanglements for *E*_eff_. When the micro-pores and entanglements of the suspension are properly averaged and pre-conditioned, *F* becomes primarily controlled by *ϕ*_s,0_^44^. However, the experimentally derived ${F}_{\exp }$ relies on experimental conditions matching with the model, which are not perfect for several reasons. First, the finite length of the barrel means the entrance flow near the constriction will compromise the uniaxial assumption. Second, the actual velocity of the fibers in the nozzle can be smaller than *χv*_a_, especially when *χϕ*_s,0_ ~ 1 where volume exclusion occurs. Third, we assume the entangled fiber suspension deforms linearly (Eq. (7)) throughout the extrusion process, which may not be valid at large deformations. Despite these challenges, ${F}_{\exp }$ provides essential information on the suspension properties for the prediction of the extrusion time and extrudate concentration.

### Tuning extrudate volume fraction in entangled and free suspensions

In the extrusion of particulate suspensions, the relative motion of solid and liquid typically reduces the volume fraction of the solid content in the extrudates^7–10,35–41^. The driving forces are the hydrodynamic and frictional interactions among the particles and the wall^45^. In Fig. 4c, we show representative results from the literature on the extrudate volume fractions of particulate pastes, where all the experiments were performed with spherical or near-spherical particles made of ceramics^35–40^ and polymers^7–10,41^. Except when there is leakage in the channel^39^, all the results from the extrusion experiments produced extrudates with equal or lower solid content than the input suspension. Similar observations were also made in our experiments with free straight fibers with aspect ratio 72, which corresponds to free suspensions where *E*_eff_ → 0 and thus ${\overline{v}}_{a}\to \infty$.

Extrudates with significantly higher volume fractions than the initial volume fractions are possible for entangled suspensions. Using a syringe with *χ* = 20, the ratio *ϕ*_s,ex_/*ϕ*_s,0_ of looped fibers ranges from 6.8 to 14, corresponding to ${\overline{v}}_{a}$ between 0.05 to 0.39 (Fig. 4c).

The ratio *ϕ*_s,ex_/*ϕ*_s,0_ of an entangled suspension can thus be controlled by varying the channel geometry *χ* and fluid-solid interactions through the parameter ${\overline{v}}_{a}$ (Fig. 4d). ${\overline{v}}_{a}$ can be adjusted by either varying the flow time scale, i.e., *L*/*v*_a_, or the poroelastic response of the suspension through *T*_pe_ (in particular liquid viscosity, fiber shape, and flexibility). In the limit of small ${\overline{v}}_{a},{\phi }_{{{{{{{{\rm{s,ex}}}}}}}}}/{\phi }_{{{{{{{{\rm{s}}}}}}}},0}\approx \chi$ indicating a stiff entangled network or weak hydrodynamic interactions. In the limit of large ${\overline{v}}_{a},{\phi }_{{{{{{{{\rm{s,ex}}}}}}}}}/{\phi }_{{{{{{{{\rm{s}}}}}}}},0}\approx 1$, indicating a soft entangled network and strong hydrodynamic interactions. Large ${\overline{v}}_{a}$ often results in large deformation of the network that could lead to break down of the linear stress response (Eq. (7)) or total break up of the network^46^. For a given entangled suspension, once the value of ${F}_{\exp }$ is obtained through a set of calibration experiments, the extrudate concentration can be tuned by adjusting the flow time scale *L*/*v*_a_ and the channel geometry *χ*.

Compared to other entangled materials, such as nanofibrillated cellulose^7^, microtubules^25^ and sticky particles^24^, the entangled microfibers are distinct in their combined properties of high permeability and strong entanglements, i.e., low ${\overline{v}}_{a}$. Such properties preserve the entangled network as a whole as the suspension passes a constriction. The mechanism and method for controlling the volume fraction variations of entangled suspensions in this work thus pave the way for future explorations of using these materials in healthcare, three-dimensional printing, and material repair.

## Methods

### Jet assisted wet spinning (JAWS) fiber synthesis

Poly(ethylene glycol) diacrylate (PEGDA) fibers were prepared in a jet assisted wet spinning (Supplementary Fig. S1) setup. The assembly of the needles in JAWS was made with a 27 gauge (27G) needle bent to be within 2 mm distance to the end of a 34 gauge (34G) needle (Cellink, MA). The ends of both needles were immersed and placed near one side of a water-filled tank (9 cm by 9 cm in width and 12 cm in height). For making straight fibers, the position of the needle assembly was fixed. For making looped fibers, the needle assembly was mounted on a mechanical vibration generator (PASCO Scientific, CA). The vibration generator oscillates horizontally at frequencies of 60, 30, and 15 Hz for producing, respectively, 4-, 2- and 1-looped fibers. The oligomer solution was composed of 80 vol % PEG-diacrylate (PEGDA, molecular weight = 575 g mol^−1^), 16 vol % deionized (DI) water and 4 vol % 2-hydroxy-2-methylpropiophenone (photoinitiator). Less than 1 vol % reactive dye, acryloxyethyl thiocarbomoyl rhodamine B (Polysciences), was added to aid in visualizing the fibers. Water was supplied through the 34G needle at a constant flow rate of 0.5 ml min^−1^ and the oligomer solution was supplied through the 27G needle at a constant flow rate of 5 μl min^−1^, using syringe pumps (Harvard Apparatus). Unless otherwise stated, all chemicals were purchased from Sigma-Aldrich.

UV light was used to initiate the cross-linking reaction in the monomer jet. The UV light was supplied by a 365 nm LED light source (M365LP1, Thorlabs) focused through an objective to a 1 mm by 1 mm region. To make straight fibers, 60 ms ON and 40 ms OFF times or 550 ms ON and 50 ms OFF times of the UV light were used for fibers of aspect ratio (AS) 72 and 360, respectively. To make looped fibers, 60 ms ON and 40 ms OFF times were used.

### Extrusion visualization setup

The polydimethylsiloxane (PDMS) (Dow Sylgard 184) channels in Fig. 1 were plasma bonded to glass slides using a Corona Surface Treater (Electro-Technic Products, Inc.). The PDMS was formed on an acrylic mold milled by a CNC machine (Bantam Tool). The channel has a uniform depth of 8 mm, a width of 10 mm in the barrel section, and a width of 2 mm in the nozzle section, thus the ratio between the cross sectional areas of the barrel (*A*_b_) and the nozzle (*A*_n_) yield *χ* ≡ *A*_b_/*A*_n_ = 5. The barrel section has a total length of 45 mm and opens to atmosphere. The nozzle section has a total length of 40 mm. Before the experiments, the channel was placed vertically on a 5 ml HSW syringe mounted on a syringe pump (Harvard Apparatus). The fiber suspension was then poured into the channel and allowed to relax for 1 min before a withdrawal flow rate of 8 ml/min was applied. Within the time of the experiment, the gravitational settling of the suspension is negligible. Videos were taken with a DLSR camera (lens: Nikon Micro-Nikkor 85mm F/3.5) and recorded on a computer.

### Extrusion experiments for *E*_eff_

For the experimental results presented in Fig. 4a for the fiber suspensions, we used standard 5 and 3 ml syringes and modified 5 and 3 ml syringes (Norm-Ject, Luer Lock). Specific parameters can be found in Supplementary Table S1. In modified syringes, the Luer section was cut and the opening was enlarged to a diameter of 4.2 mm. Before an extrusion experiment, the plunger was removed to pour the fiber suspension into the syringe. Next, the plunger was put back and adjusted to the desired volume. To accommodate the long barrel approximation in the poroelastic model, we ensured that the suspension occupied a length *L* > 3.5*D*.

### Characterization of the physical properties of fibers and fiber suspensions

The width and length of fibers are measured using dilute fiber suspensions and microscope images using ImageJ software (ImageJ version 1.53e; NIH). The length and diameter of the fibers are taken as the average of approximately 50 fibers. The straight fibers with aspect ratio (AS) of 72 have length *l* = 4.3 ± 0.7 mm and diameter *d* = 59.6 ± 2.0 μm; the straight fibers with AS of 360 have length *l* = 22 ± 1.2 mm and diameter *d* = 60.4 ± 1.7 μm. The looped fibers have *l* = 4.5 ± 0.8 mm and the loops on the looped fibers have a typical dimension of 204 ± 42 μm. The Young’s modulus, *E*_*y*_, of the polymerized PEG was measured with a tensile test below a balance (Mettler Toledo, OH), where a straight fiber was immersed in water and pulled at a constant rate of 40 μm/s. The measured *E*_y_ was in the range of 100–300 kPa for five fibers that were measured.

The pull-out experiments from fiber suspensions were conducted with a 1 mm diameter glass rod made from an end-melted glass capillary (WPI, FL) (Supplementary Fig. S4). The rod was attached to the measurement hook below a balance (Mettler Toledo, OH) connected to a computer. The end of the rod had a drop of liquid epoxy and was immersed in a water bath (10 cm by 10 cm by 30 cm). A 5 ml syringe (VWR) was prepared with the Luer slip cut off and filled with 3 ml of fiber suspension. The syringe was moved up from directly underneath the rod until the epoxy touched the suspension. The epoxy was then cured with UV light to attach the fibers to the rod. The rod did not touch the syringe throughout the process. The pull-out experiment was carried out by moving the syringe down using a linear translation stage (NRT100, Thorlabs) at a constant velocity of 40 μm/s.

### Rheological measurement of fibers suspensions

Rheological measurements were conducted on an Anton Paar MCR 301 rheometer with a 50 mm diameter sand-blasted parallel plate. The gap between the parallel plates was 1.0 mm. ${G}^{{\prime} }$ at a shear strain of 1% was obtained from oscillatory shear measurements using a frequency of 1.6 Hz.

## Supplementary information

Supplementary Information

## Data Availability

The datasets generated during and/or analysed during the current study are available in the Zenodo repository, 10.5281/zenodo.7637463.

## Code availability

All custom codes used for data preparation are deposited online at 10.5281/zenodo.7637463.
