# Supplementary material for: Controlling extrudate volume fraction through poroelastic extrusion of entangled looped fibers
Source: Nat Commun. 2023 Mar 4;14:1242. doi: 10.1038/s41467-023-36860-y (PMC9985605; doi:10.1038/s41467-023-36860-y)
Supplement: Supplementary file 1 — Supplementary Information [file 41467_2023_36860_MOESM1_ESM.pdf]

SUPPLEMENTARY INFORMATION

Controlling Extrudate Volume Fraction through Poroelastic Extrusion of Entangled Looped Fibers

Zehao Pan[1], Janine K. Nunes[1], Camille Duprat[2], Ho Cheung Shum[3] and Howard A. Stone[1*]

[1]Department of Mechanical and Aerospace Engineering, Princeton University, Princeton, NJ 08544.
[2]LadHyX - École Polytechnique, Palaiseau Cedex 91128, France.
[3]Department of Mechanical Engineering, University of Hong Kong, Pokfulam Road, Hong Kong, 999077 China.

*Corresponding author(s). E-mail(s): hastone@princeton.edu;

Inventory of Supplementary Information:

Supplementary Figure S1. JAWS
Supplementary Figure S2. Microscopic images of entanglement.
Supplementary Figure S3. Extrusion regimes.
Supplementary Figure S4. Pull-out measurements.
Supplementary Figure S5. Additional simulation results.
Supplementary Figure S6. Extrusion of looped fibers using syringes.
Supplementary Table S1. Experimental conditions for extrusion.
Supplementary Figure S7. Rheology of fiber suspensions.
References

We used jet assisted wet spinning (JAWS) to fabricate straight and looped poly(ethylene glycol) (PEG) microfibers from light-activated gelation chemistry. In JAWS, a slower monomer jet in a water bath is accelerated and thinned by a faster water jet (Supplementary Figure S1(a)). The ultraviolet (UV) light is introduced 2 cm downstream of the water jet needle to crosslink the monomer. When making looped fibers, horizontal oscillation is added to both the water jet and the monomer jet. Under the oscillation, the monomer jet not only acquires horizontal displacement but also adopts a different speed from the water jet [1]. As the monomer jet moves downstream, the speed difference develops into a vertical displacement difference. Together with the horizontal displacement from the oscillation, a looped structure is formed, which is retained permanently in the fibers by UV polymerization.

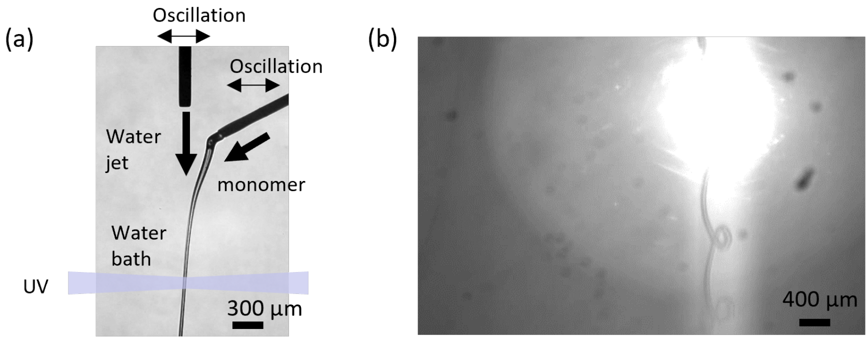

Fig. S1 Jet assisted wet spinning (JAWS). (a) Snapshot for JAWS. A monomer jet in a water bath is accelerated and thinned by a water jet. UV light is introduced downstream to crosslink the monomer. When making looped fibers, horizontal oscillation is added to both water jet and monomer jet. (b) Snapshot showing looped fibers being produced. The bright spot is the UV light spot.

At rest, the looped fiber suspension exhibits random orientations and separations as shown in Supplementary Figure S2(a). Upon stretching a single fiber, temporary mechanical links are created among nearby fibers that can pass on tension, as shown in Supplementary Figure S2(b). The nearby fibers respond by reorientation and translation which passes on the stress to the fibers further away. Under confinement, the limited separations among the fibers facilitate the formation of the temporary mechanical links. As a result, large elastic deformations can be induced in the entangled suspension through reorientation and translation of the fibers.

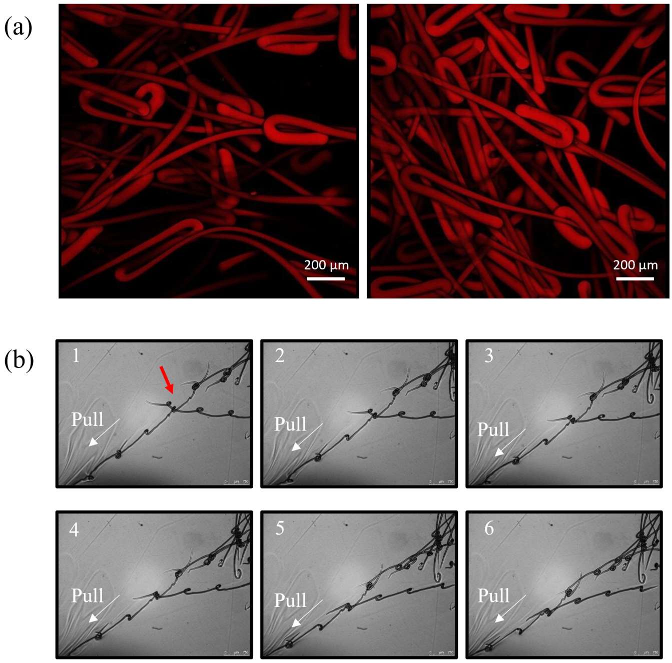

Fig. S2 (a) Confocal images of a suspension of looped fibers. (b) Time series (1-6) of an entanglement forming. A fiber is pulled to the left bottom corner while hooking with another fiber through the loop structure interaction, as indicated by the red arrow.

Depending on the degree of entanglements before extrusion, the fiber extrudate can form an integral entangled 'gel' or remain dispersed and fragmented. We investigated the effect of fiber geometry and volume fraction $\phi_{s,0}$ on the morphology of the extrudate. We used a syringe with $\chi = 12$ with initial volume of 3 ml. For straight fibers we kept the diameter d constant at 60 μm and change the length l. When the l/d is 166, the extrudate is dispersed similar to its state before extrusion. When the l/d is 250, the extrudate is fragmented but more concentrated. We find the extrudate can form an integral 'gel' when the aspect ratio l/d is 360 at volume fractions from 0.04 to 0.2 as shown in Supplementary Figure S3(a). At this aspect ratio, the fiber length l, however, is more than 50% of the total barrel length L ($l/L = 0.55$). Thus, we conclude that for the extrusion conditions investigated, straight flexible fibers are not able to create an integral 'gel' without its length being close to L. It is then unsuitable for straight flexible fibers to be considered by a poroelastic model based on continuum assumption in the main text. According to the simulation of similar athermal straight fibers [2], the weak entanglements could be attributed to low friction among the fibers, fibers with too much stiffness or a lack of interwoven structure in the network topology.

For looped fibers, more than one loop is required for forming an extrudate 'gel'. Relative to the 4-looped fibers, the 2-looped fibers require slightly higher volume fraction to achieve enough entanglements for forming an integral extrudate 'gel', as shown in Supplementary Figure S3(b).

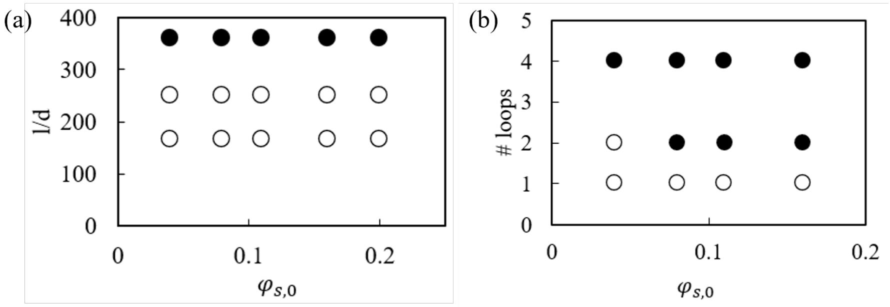

Fig. S3 Regimes for extrusion of entangled fiber suspension as a function of volume fraction $\phi_{s,0}$ and fiber geometry: (a) straight fibers with three aspect ratios l/d; (b) looped fibers with 1, 2 and 4 loops per fiber. In the conditions indicated by the filled symbols, the extrudate forms an integral 'gel', while the closed symbols indicate the extrudates are dispersed or fragmented. All extrusion starts at 3 ml, at a flow rate of 5 ml/min with $\chi = 12$.

We used a pull out test to estimate the elastic modulus E_{eff} of an entangled fiber suspension. The setup and schematic of the pull-out test is shown in Supplementary Figure S4(a) and (b). The stress is defined as the pull-out force divided by the cross sectional area of the probe, i.e., $F/\pi r^2$. The strain is defined as the probe displacement divided by the probe diameter $2r$. The number of fibers being pulled out is between 5 and 12, but the stress and strain relationships are similar among different tests for the same suspension. Three typical stress-strain relationships are shown in Supplementary Figure S4(c) for 4-looped fibers at $\phi_{s,0} = 0.15$. While we can not extract quantitative values of modulus using these data, the stress and strain relationship allows the estimation of E_{eff}, on the order of 10^1 to 10^2 Pa at strain of 100%.

In comparison, at the same $\phi_{s,0}$ and similar fiber aspect ratio at 72, the straight fibers show negligible elastic response during the pull out test. At much higher aspect ratio of 360, the straight fibers shows some elastic response but still smaller than the looped fibers at the same $\phi_{s,0}$, revealing the significance of the looped shape in enhancing the entanglements of the fiber suspension.

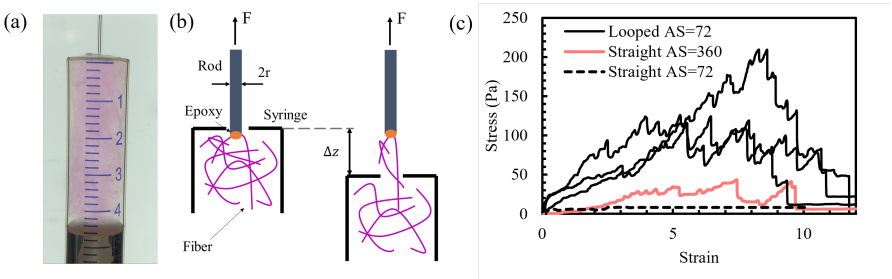

Fig. S4 (a) Snapshot of pull-out test of the looped fiber suspension (see *Materials and Methods* section for details). (b) Schematic of the pull out test. (c) Stress strain relationship of the pull out test of suspensions of looped and straight fibers. 'AS' stands for aspect ratio. All curves are measured at solid volume fraction $\phi_{s,0}$ of 0.15. The looped ($AS = 75$) and straight ($AS = 72$) fibers are used in Figure 1 in the main text.

During poroelastic extrusion, the entangled fiber network stretches and dilutes the local solid volume fraction. Supplementary Figure S5(a) shows the solid volume fraction variations during the extrusion of looped fibers under the same condition as Figure 1 in the main text. The dilution in the solid volume fraction ϕ_s (increase in ϕ_f) begins from $x = L$ and expands to $x = \delta$. A maximum is reached in in the middle of the extrusion process. In the Eulerian frame, the maximum in internal elastic stress is readily observable in Supplementary Figure S5(b). The highest level of stretching elastic stress among the fiber network occurs at the constriction. The stress disappears at free end of the suspension at $x = \delta$.

The extrudate volume fraction, presented as the the ratio $\phi_{s,ex}/\phi_{s,0}$, is predominantly determined by χ and $\overline{v}_a$. To fully describe the poroelastic model, $\phi_{s,0}$ also needs to be specified. We show the effect of $\phi_{s,0}$ in Supplementary Figure S5(c). The effect of $\phi_{s,0}$ on $\phi_{s,ex}/\phi_{s,0}$ is smaller than 4% for the two cases $\chi = 2$ and $\chi = 9$ across three decades of $\overline{v}_a$.

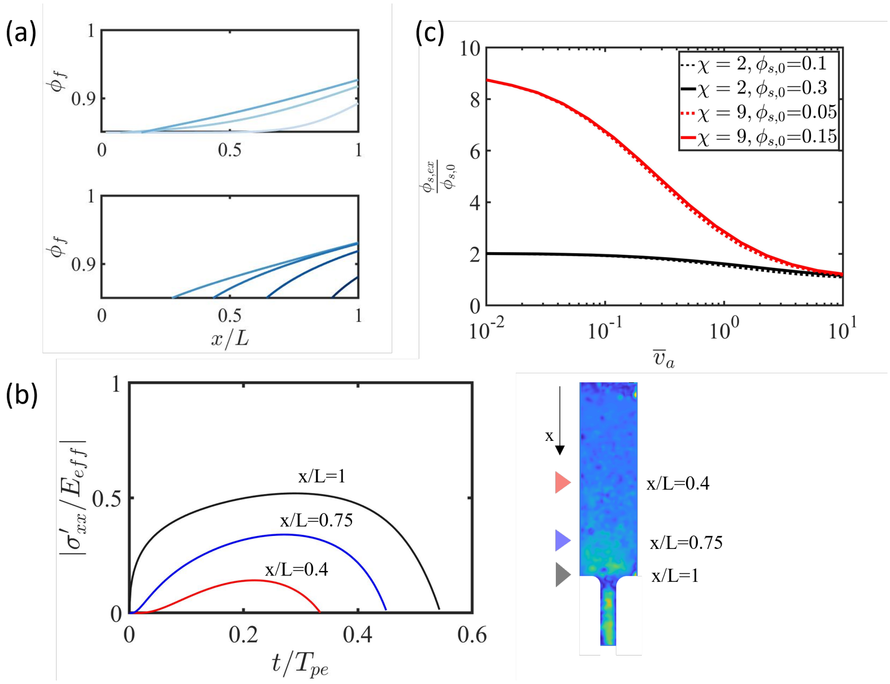

Fig. S5 (a) The volume fraction variation at the same time stamps as in Figure 3(d) in the main text. The time increases from lighter blue to darker blue. (b) The σ'_{xx} at locations in the extrusion channel labeled by corresponding colored triangles.(c) The simulated $\phi_{s,ex}/\phi_{s,0}$ at different value of $\phi_{s,0}$ and $\overline{v}_a$ at $\chi = 2$ and $\chi = 9$.

Supplementary Figure S6(a) shows the typical time sequence of an entangled suspension being extruded out of a syringe. The extrudate forms an integral 'gel' (the long magenta bundle outside the syringe) when the degree of entanglement is high in the suspension. Supplementary Figure S6(b) shows the commercial and modified syringes used that represent different constriction ratios. The detailed experimental conditions in the main text is shown in Supplementary Table S1.

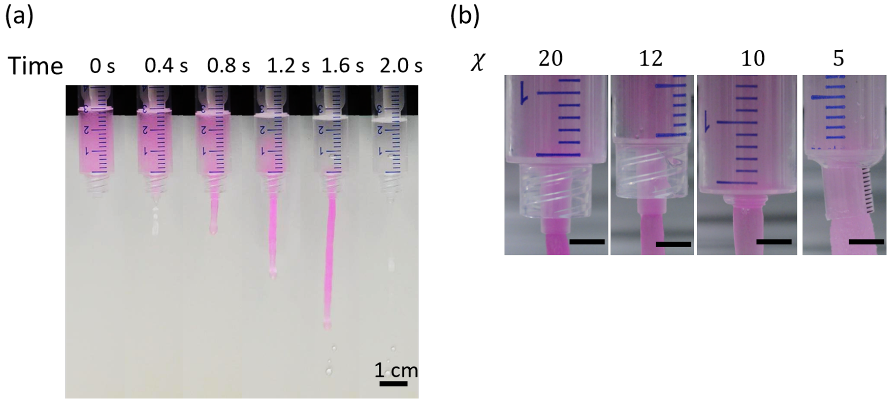

Fig. S6 (a) Typical time sequence when entangled fibers (looped) are extruded through a syringe. The magenta fibers started as distributed throughout the syringe. During extrusion the fibers expelled water from the suspension while being concentrated. (b) Snapshots during the extrusion of looped fibers using different extrusion geometries for data in Figure 4(b) in the main text. The scale bar is 500 μm.

Table S1 Experimental conditions and $\phi_{s,ex}$ for data in Figure 4 in the main text.

χ	A_b (mm^2)	L (mm)	Q_0 (ml/min)	$\phi_{s,0}$	$\phi_{s,ex}/\phi_{s,0}$
20	122.7	48.9	8	0.062	11.8±0.8
20	122.7	48.9	8	0.093	10.0±0.5
20	122.7	40.7	8	0.036	12.8±0.7
20	122.7	48.9	8	0.047	12.7±0.8
12	72.4	41.4	5	0.12	6.5±0.5
12	72.4	41.4	5	0.06	7.0±0.7
12	72.4	41.4	5	0.03	10.0±0.8
10	122.7	40.7	8	0.074	6.3±0.2
10	122.7	40.7	8	0.11	5.3±0.4
5	72.4	41.4	5	0.19	2.5±0.2

The yield stress and shear modulus of the fiber suspensions are characterized in a rheometer (Supplementary Figure S7(a)). By applying a constant shear stress on a suspension the suspension either deforms continuously over time (more than 5 minutes) or stops deforming after a finite time as shown in Supplementary Figure S7(b). We refer to the previous case as the yielded case and the latter case as the not yielded case. We adjust the applied stress to narrow the stress between the two cases until the difference is smaller than 30%. The yield stress is calculated between the two nearest yielded and not yielded cases (Supplementary Figure S7(c)).

The storage modulus of the suspension of looped fibers is measured on a rheometer using amplitude sweep as shown in Supplementary Figure S7(d). The storage modulus is higher than the loss modulus when the strain amplitude is smaller than 40 % to 100 %. As a function of the volume fraction, the storage modulus shows a power law relationship with an exponent of 2.95 in Supplementary Figure S7(e).

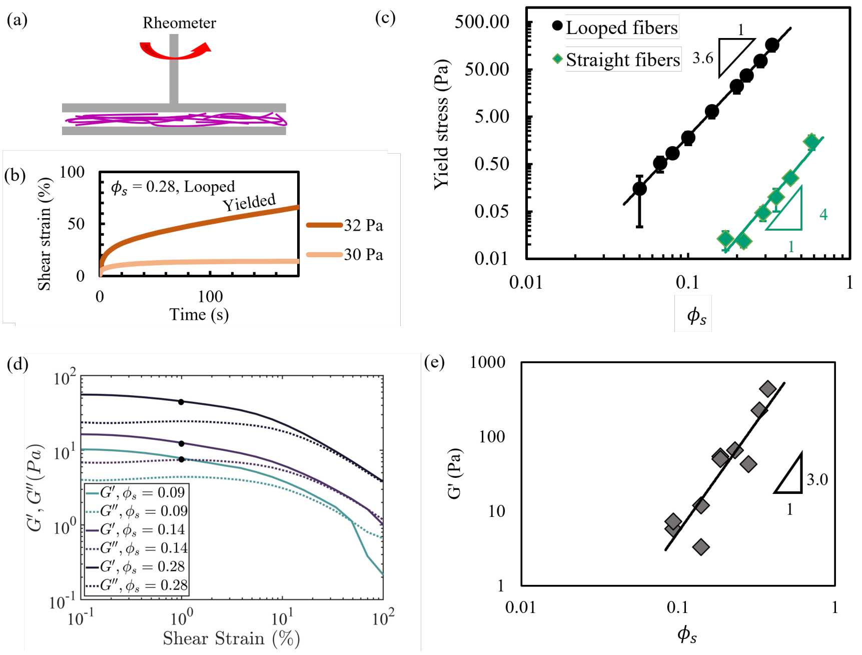

Fig. S7 (a) Rheology tests are performed with a sandblasted 50 mm parallel plate. The gap between the plates is 1 mm for all tests. (b) Strain response under constant shear stress of a looped fiber suspension at solid volume fraction of 0.28. When the applied stress is higher than the yield stress the shear strain will increase indefinitely (longer than 5 minutes in the test). (c) Yield stress of the entangled suspension made from looped fibers ($AS = 75$) and free suspension made from straight fibers ($AS = 72$) as a function of the solid volume fraction. (d) Storage (G') and Loss (G") moduli of looped fiber suspension at different solid volume fractions $\phi_{s,0}$. The oscillatory frequency is 10 rad/s for all amplitude sweeps. (e) G' at 1% shear strain (dots in (d)) as a function of $\phi_{s,0}$.

References

[1] Pope, S. B. *Turbulent flows* (Cambridge university press, 2000).

[2] Negi, V. & Picu, R. Tensile behavior of non-crosslinked networks of athermal fibers in the presence of entanglements and friction. *Soft Matter* **17** (45), 10186–10197 (2021) .
